# Spect perfusion imaging versus CT for predicting radiation injury to normal lung in lung cancer patients

**DOI:** 10.1259/bjr.20190184

**Published:** 2019-07-05

**Authors:** Alex Weller, Alex Dunlop, Adam Oxer, Ranga Gunapala, Iain Murray, Matthew J Gray, Glenn D Flux, Nandita M deSouza, Merina Ahmed

**Affiliations:** 1 The CRUK Cancer Imaging Centre, The Institute of Cancer Research and The Royal Marsden Hospital NHS Foundation Trust, Sutton, Surrey; 2 Department of Radiology, Northwick Park Hospital, Watford Road, Harrow, London; 3 The Joint Department of Physics, The Royal Marsden Hospital NHS Foundation Trust and The Institute of Cancer Research, Sutton, Surrey; 4 The Royal Marsden Hospital NHS Foundation Trust, Sutton, Surrey

## Abstract

**Objectives:**

In non-small cell lung cancer (NSCLC) patients, to establish whether the fractional volumes of irradiated anatomic or perfused lung differed between those with and without deteriorating lung function or radiation associated lung injury (RALI).

**Methods:**

48 patients undergoing radical radiotherapy for NSCLC had a radiotherapy-planning CT scan and singlephoton emission CT lung perfusion imaging (^99m^Tc-labelled macroaggregate albumin). CT defined the anatomic and the singlephoton emission CT scan (co-registered with CT) identified the perfused (threshold 20 % of maximum) lung volumes. Fractional volumes of anatomic and perfused lung receiving more than 5, 10, 13, 20, 30, 40, 50 Gy were compared between patients with deteriorating (>median decline) *vs* stable (<median decline) forced expiratory volume in 1 s (FEV1) and between those with and without RALI (assessed by Common Toxic Criteria for Adverse Events) radiation pneumonitis and pulmonary fibrosis scores.

**Results:**

Fractional volumes of anatomic and perfused lung receiving more than 10, 13 and 20 Gy were significantly higher in patients with deteriorating *vs* stable FEV1 ( *p* = 0.005, 0.005 and 0.025 respectively) but did not differ for higher doses of radiation (>30, 40, 50 Gy). Fractional volumes of anatomic and perfused lung receiving > 10 Gy best predicted decline in FEV1 (Area under receiver operating characteristic curve (Az = 0.77 and 0.76 respectively); sensitivity/specificity 75%/81 and 80%/71%) for a 32.7% anatomic and 33.5% perfused volume cut-off. Irradiating an anatomic fractional volume of 4.7% to > 50 Gy had a sensitivity/specificity of 83%/89 % for indicating RALI (Az = 0.83).

**Conclusion:**

A 10–20 Gy radiation dose to anatomic or perfused lung results in decline in FEV1. A fractional anatomic volume of >5% receiving >50 Gy influences development of RALI.

**Advances in knowledge:**

Extent of low-dose radiation to normal lung influences functional respiratory decline.

## Introduction

In patients with non-smallcell lung cancer (NSCLC), administration of curative radiation therapy (RT) results in radiation associated lung injury (RALI), which consists of radiation pneumonitis in the acute phase and pulmonary fibrosis in the late phase.^1^ Where underlying lung function is abnormal, RALI risks vital loss of respiratory function and the morbidity of debilitating dyspnoea. Therefore, escalation of radiation dose to increase the probability of tumour cell death also must be traded against the rising probability of RALI.^2^ Although regional variation in lung function^3^ is documented, treatment planning simplistically considers anatomic lung as a uniform structure. This is because the incidence of symptomatic early phase radiation pneumonitis and later pulmonary fibrosis increase with the anatomic volume of lung that is irradiated and the dose (in Gy) delivered^4^; mean lung dose (MLD) and the fractional volume (%) of lungs receiving greater than an indicated dose are predictive of RALI.^1,5,6^

More recently, functional image-guided RT planning for normal lung avoidance using single photon emission CT (SPECT),^7^ 18-fluodeoxy glucose positron emission tomography (^18^FDG PET) or hyperpolarized gas MRI have been advocated to avoid dose delivery to normal lung.^8–13^ Although SPECT perfusion studies have long been investigated as predictor of RALI, perfusion-weighted threshold values have varied between studies and confirmatory data is required.^7,9,14–18^ ^99m^Tc labelled macroalbumin aggregate (MAA) SPECT imaging (using aggregates 10–150 µm in diameter that accumulate in pulmonary capillaries with a regional concentration that is proportional to first pass pulmonary blood flow) provides a quantitative measure of regional variation in pulmonary perfusion.^19–21^ The technique may be exploited to assess toxicity based on radiation dose to perfused rather than anatomic lung. Two studies have specifically compared functional SPECT imaging with conventional CT imaging for predicting RALI.^7,22^ Both showed that functional SPECT imaging could better predict radiation pneumonitis in lung cancer patients treated with curative radiotherapy, although the differences between predictive metrics did not reach statistical significance. This study therefore aimed to establish whether fractional volumes of irradiated perfused lung identified on SPECT imaging differed between those with and without deteriorating lung function (as measured by forced expiratory volume at 1 s, FEV1) or RALI (using Common Toxic Criteria for Adverse Events (CTCAE) radiation pneumonitis and pulmonary fibrosis scores).^23^ The findings were compared with equivalent data for irradiated fractional anatomic lung volumes.

## Methods

Between 18 May 2010 and 26 May 2015, this prospective, institutional review board approved, single-centre trial recruited 58 patients aged 45–87 years old (Table 1). All gave written informed consent. Patients had histologically confirmed locally advanced non-resectable NSCLC (all subtypes) suitable for radical radiotherapy (Stages I–IIIb). 10 were withdrawn prior to radiotherapy (6 changed treatment plan, 1 opted for surgery prior to radiotherapy, 1 unable to tolerate the study imaging, 1 withdrew consent, 1 missed the SPECT perfusion scan appointment). The 48 patients enrolled underwent a planning CT thorax scan (thoracic inlet to below the diaphragm) with intravenous contrast as clinically indicated. The scan was performed in breath-hold with the use of the an active breathing control (ABC) device,^24^ or a four-dimensional (4D) scan was acquired in free-breathing if the ABC device was not tolerated. Within a 4 h period, the patient underwent a lung SPECT perfusion scan.

**Table 1.** Patient characteristics for *n* = 48 patients undergoing ^99m^Tc-MAA SPECT perfusion prior to radiotherapy, documenting demographics, tumour location, lung volumes and planning target volumes

**Characteristic**	**SPECT CT pre RT, plus CTCAE (*n* = 44) or FEV1 (*n* = 41) outcome data**
**Age (yr)**	
Mean [sd; range]	68 [9.1; 45–81]
**Sex**	
M	22
F	21
**Clinical stage**	
I-II	14
IIIA-IIIB	29
**Histology**	
Adenocarcinoma	18
Squamous cell carcinoma	18
Unspecified NSCLC, large cell or not recorded	6
**WHO PS at baseline**	
0	9
1	34
**Smoking status**	
Current smoker	17
Previous smoker (never smoked)	26 (2 never smoked)
**Tumour location**	
Upper lobe	25
Middle lobe or lingula	10
Lower lobe	8
**RT volumes**	
PTV (cm^3^) mean [sd; range]	167.3 [116.2; 23–757.5]
Total lung volume (cm^3^) median [sd; range]	4790 [1279; 2638–7735]

CTCAE, Common Toxic Criteria for Adverse Events; MAA,macroalbumin aggregate; NSCLC, non-small cell lung cancer; PTV, planning target volume; RT, radiation therapy; sd,standard deviation.

RT planning was routine; patients received either conformal or intensity modulated RT as clinically indicated (60–66 Gy in 2 Gy fractions over 6.5 weeks) or accelerated hypofractionated regimen (55 Gy in 20 daily fractions over 4 weeks). Gross target volume (GTV) or internal target volumes (ITVs) were created for ABC and 4DCT scans respectively. For the 4DCT, the maximum inhale and exhale phases (0 and 50% as defined with our CT scanner) were used for GTV definition. These were then combined on the average CT plan to generate an ITV. Treatment planning was performed on the 4DCT average scan reconstruction. Anatomic lung volumes were delineated as a single volume using CT lung windows to ensure inclusion of all parenchyma from apices to bases, including regions of collapse or consolidation and ensuring that the GTV/ITV, trachea and proximal bronchial tree were excluded from this volume. No clinical target volume (CTV) margin was required and GTV-planning target volumes (PTVs) margins for ABC scans were circumferentially 1 cm. For 4DCT scans, margins for ITV to PTV were 0.5 mm axially and 0.7 mm craniocaudally. Three-dimensional conformal plans were generated using the Pinnacle3 treatment planning system (Philips Medical Systems, Best, NL), using a collapsed cone convolution calculation algorithm for dose calculation. All plans were optimized to comply with the following dose-volume constraints: MLD <20 Gy; V20 <35%; and in some cases V5 <60%. This was performed without reference to the functional SPECT data.

41 patients had FEV1 and forced vital capacity (FVC) measured at least once following therapy (no spirometry data were recorded in the other 7) and 40 patients had CTCAE recorded at either 3 or 6 months post-RT. The eight patients with missing CTCAE data at 3 and 6 months comprised 5 who died, one who had surgery within 1 month of radiotherapy and one patient who missed their trial follow-up appointments.

## SPECT scanning and image Post-processing protocols

Patients were positioned in the radiotherapy position (supine with arms above their heads), on the γ camera couch, prior to intravenous injection of 200 megabecquerels (MBq) of 99mTc-MAA. SPECT images were acquired with a dual-headed γ camera (Phillips Medical Systems Forte or Siemens Intevo), with a low energy high resolution collimator at discrete 3° angular intervals with each camera head rotating through 180° (64 projections at 20 s per camera view using a 128 × 128 matrix, pixel size 4.67 mm). Data were reconstructed using the ordered subset expectation maximization (OSEM) algorithm on a clinical workstation (Hermes Medical Solutions Incorporated, Stockholm, Sweden).

SPECT perfusion data were anatomically co-registered and fused with the radiotherapy planning CT (Syngo, Siemens Medical Solutions, Forchheim, Germany). Automated image anatomic co-registration was applied. Mis-registrations were manually adjusted by an experienced radiologist (AW) and took into account differences in breath-hold between SPECT (free breathing) and RT planning (breath-hold in moderate deep inspiration inspiration). Where discrepancy existed in the level of pulmonary inflation between the RT planning CT and SPECT images, the lung apices, the hilae and any perfusion defect associated with the primary tumour were used as reference points. These represented structures that were either relatively fixed in position with inspiration (lung apices in contra distinction to the diaphragm), or were unlikely to contain radiotracer activity due to first-pass uptake within the pulmonary capillary bed (*e.g.* tumour and hilar structures).

**Figure 1.**
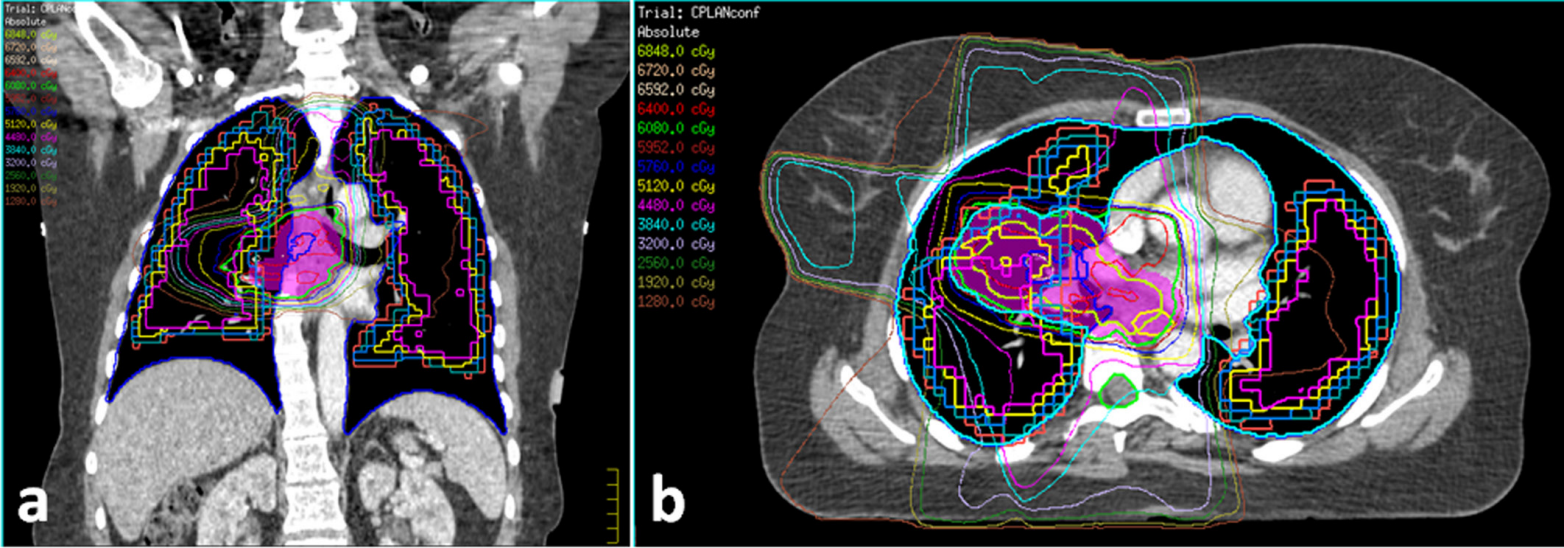
Functional perfusion volumes and RT isodose contours: axial (a) and coronal (b) images through a right hilar tumour. The dark blue volume encompassing both lungs represents anatomical lung volume ("lung volume – PTV"). The smaller volumes contained within this represent the "perfusion volumes": Red = p20 (voxels with ≥20% of maximum voxel intensity); Green = p25; Pale blue = p30; Yellow = p40; Magenta = p50. The RT iso-dose contours are overlaid on these volumes to demonstrate the dose delivered to a right hilar and upper lobe tumour in this patient. PTV contour is not included in this image. PTV,planning target volumes; RT, radiation therapy.

"Perfusion volumes" were defined using a two-step process. First, all voxels within both lungs that contained tracer activity were contoured within a single boundary. Second, "perfusion volumes" were generated within this boundary by setting a threshold constraint to include only those voxels that contained equal to or more than a defined proportion of maximum voxel perfusion count. A 20% threshold of maximum voxel perfusion count (p20) was used.^14^ At higher perfusion thresholds (25, 30, 40 and 50% of maximum voxel perfusion count), discrepancies between anatomic and perfused lung volumes as a result of differences in levels of inspiration during data acquisition led to more severe SPECT/planning CT image mis-registration. As reported by prior groups, p20 was therefore selected as the most robust measure of perfused lung. Perfusion volumes generated using the co-registered SPECT and RT planning data sets were imported into the Pinnacle3 RT-treatment planning platform (Figure 1).

Dose–volume histograms were calculated for volume of anatomic (a) and perfused (p20) lung receiving more than 5, 10, 13, 20, 30, 40, 50 Gy and MLD (denoted as aV5, aV13, aV20, aV30, aV40, aV50, aMLD and pV5, pV10, pV13, pV20, pV30, pV40, pV50, pMLD respectively).

## Clinical assessments

Clinical assessment was carried out at baseline and 1, 3, 6, 9, 12, 15, 18 and 24 months post-treatment using spirometry (FEV1). Radiation associated lung injury was assessed at all follow-up time-points, using the CTCAE v. 3.0 grading system for characterizing radiation induced pneumonitis (early) or pulmonary fibrosis (late), depending on the timing of follow-up assessment. In the early phase (upto and including 6 months following end of RT), CTCAE v. 3.0 radiation pneumonitis was used and in the late phase (9 months and beyond), pulmonary fibrosis scoring was applied. Radiation associated pneumonitis and fibrosis are collectively referred to as RALI.

Under CTCAE v. 3.0, radiation-induced symptoms (dyspnoea, cough, pain and low-grade fever) plus distinctive radiological changes on CT or chest X-ray were evaluated. A distinction was made between "mild" (Grade 0–1) and "moderate–severe" (Grade 2–5) radiation pneumonitis and pulmonary fibrosis, as grades ≥2 are defined by symptoms that limit activities of daily living and/or require medical intervention (early phase–radiation pneumonitis) and moderate hypoxaemia (late phase–pulmonary fibrosis). Worst CTCAE radiation pneumonitis grade was recorded at 3 and 6 months following RT (when RT-induced *pneumonitis* is expected to peak). Mann–Whitney U-tests were used to assess for a difference in dose–volume parameters between "radiation pneumonitis" and "no radiation pneumonitis" groups.

## Data analysis

Data were analysed in Excel and Graph-pad Prism (v. 8). A dose parameter of V20 was applied to SPECT-perfused lung volumes (as an independent organ-at-risk), to discriminate between "RALI" and "no RALI" groups on CTCAE v. 3.0. The fractional change in FEV1 after radiotherapy was calculated:



Greater than or less than median FEV1 fractional change discriminated patients with deteriorating *vs* stable respiratory function. Data from all follow-up visits were collated to describe "RALI," graded according to CTCAE v. 3.0—early phase radiation pneumonitis and late phase pulmonary fibrosis.

## Statistical analysis

Fractional lung volumes defined by anatomic and perfused lung receiving >5, 10, 13, 2, 30, 40 and 50 Gy were compared using one-way analysis of variance (ANOVA).

Differences in anatomic and perfused lung dose-volume parameters were evaluated between patients with Deteriorating *vs* Stable FEV1 using the independent Student’s *t*-test. Receiver operating characteristic (ROC) analyses estimated the ability of anatomical and perfused lung dose–volume parameters to predict deterioration in FEV1. Similar analyses were undertaken between those with and without radiation pneumonitis and with and without RALI (Mann–Whitney *U* statistic). Bonferroni correction was used to set the level of significance as *p* = 0.00625 (=0.05/8). This is based on the assumption of no second-order interactions, and potentially sets excessively stringent requirements for statistical significance.^25^

## Results

### Comparison of anatomic and perfused lung volumes and dose–volume parameters

**Figure 2.**
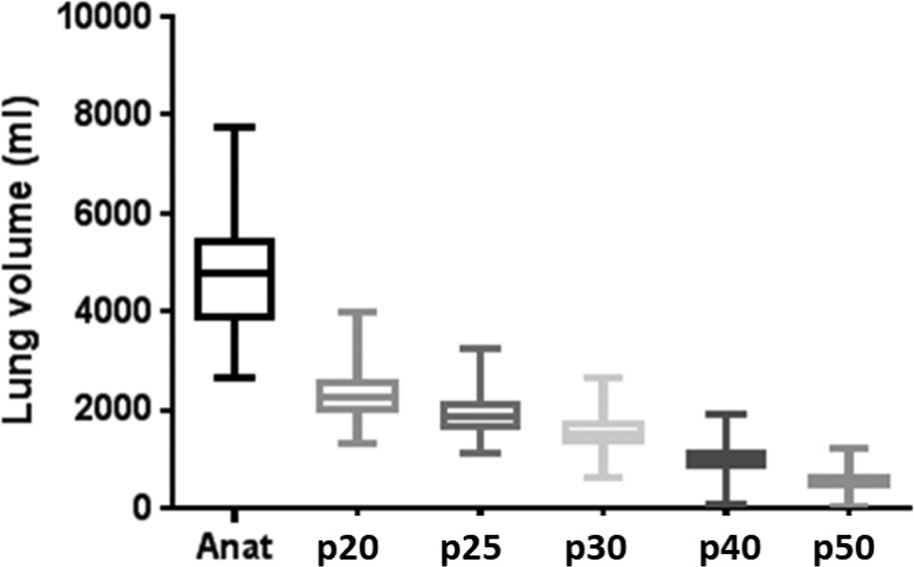
Boxplot of the anatomic and perfused lung volumes delineated using the RT planning and SPECT perfusion imaging respectively. Perfused volumes are all significantly smaller than the anatomic volumes (at all thresholds), with the biggest change occurring between the anatomic and the p20 volume. The p50 volume is the smallest for each patient. RT,radiation therapy; SPECT, single photon emission CT.

The volumes of anatomic and perfused lung receiving 5, 10, 13, 20, 30, 40 and 50 Gy dose thresholds are given in Table 2 and illustrated as boxplots in Figure 2.

**Table 2.** Absolute volumes of anatomic and perfused lung (p20) receiving ≥5, 10, 13, 20, 30, 40 and 50 Gy

**Volume**	**Anat vol (ml) mean (sd)**	**p20 vol (ml) mean (sd)**	**p25 vol (ml) mean (sd)**	**p30 vol (ml) mean (sd)**	**p40 vol (ml) mean (sd)**	**p50 vol (ml) mean (sd)**
**Total**	4723 (1244)	2331 (571)	1936 (489)	1590 (429)	1006 (316)	556 (218)
**≥5** Gy	2132 (305)	1213 (236)	1006 (207)	824 (179)	517 (125)	276 (75)
**≥10** Gy	1519 (189)	865 (153)	709 (132)	573 (113)	348 (76)	181 (43)
**≥13** Gy	1228 (131)	679 (106)	550 (89)	438 (75)	257 (48)	128 (25)
**≥20** Gy	824 (58)	442 (48)	351 (39)	273 (32)	153 (19)	73 (10)
**≥30** Gy	544 (27)	292 (24)	227 (19)	174 (15)	93 (9)	43 (4)
**≥40** Gy	342 (13)	196 (13)	150 (10)	113 (8)	59 (4)	27 (2)
**≥50** Gy	152 (3)	110 (4)	81 (3)	59 (2)	28 (1)	11 (0.4)

NB Differences between anatomic volumes and p20 volumes at all dose levels were significant (p<0.0001 after Bonferroni correction).

Pairwise differences confirmed that the anatomic volume was significantly larger than the perfused volume across the entire cohort; the biggest difference occurred between anatomic and perfused lung volumes receiving >20 Gy (aV20 *vs* pV20), for all dose thresholds, (*p* < 0.0001, Table 2).

### Effect of pulmonary dose-distribution on spirometry

Fractional volumes of both anatomic and perfused lung receiving >10 and 13 Gy were significantly larger in patients with deterioration in FEV1 (greater than median FEV1 change) compared with those who had stable FEV1 (*p* ≤ 0.005, Tables 3 and 4). This indicates that low dose radiation to both anatomic and perfused lung results in subsequent reduction in respiratory function. The fractional volumes of anatomic and perfused lung receiving higher doses of radiation (30, 40 and 50 Gy) did not differ between these groups, indicating that the higher doses of radiation closer to or within the planning target volume did not result in subsequent deterioration of FEV1. Fractional volumes of anatomic and perfused lung were not significantly different between patients with “deteriorating” FVC (greater than median FVC change) or “stable” FVC at any dose threshold.

**Table 3.** Summary of anatomic lung dose-volume parameters in patients that experienced greater than ("Deteriorating FEV1") and less than ("Stable FEV1") the median percentage decline in FEV1 at different radiotherapy dose thresholds (V5-50) and for MLD

**% Anatomic volume**	**"‘Stable FEV1" (*n* = 21)**	**"‘Deteriorating FEV1"** **(*n* = 20)**	**Difference between means (%)**	***t*-test** *p-*value	**ROC AUC** **(Az)**
**V5**	39.6	47.4	7.9	0.093	0.66
**V10**	25.9	36.7	10.8	0.005**	**0.77**
**V13**	20.8	30.1	9.3	0.005**	**0.76**
**V20**	14.5	19.5	5.1	0.025	0.70
**V30**	9.9	12.4	2.5	0.129	0.65
**V40**	6.4	7.6	1.2	0.313	0.61
**V50**	2.7	3.5	0.8	0.211	0.66
**MLD (cGy)**	897	1127	229	0.040	0.69

AUC, area under the curve; MLD, mean lung dose; ROC, receiver operating characteristic.

Results that reach statistical significance after applying the Bonferroni correction (significance level 0.05/8 = 0.00625) are marked**.

**Table 4.** Summary of functional lung dose-volume parameters in patients that experienced greater than ("Deteriorating FEV1") and less than ("Stable FEV1") the median percentage decline in FEV1 at different radiotherapy dose thresholds (V5-50) and for MLD

**% p20** **volume**	**"‘Stable FEV1" (*n* = 21)**	**"Deteriorating FEV1"** **(*n* = 20)**	**Difference between means (%)**	***t*-test** *p-*value	**ROC AUC (Az)**
**p20V5**	43.8	56.0	12.2	0.050	0.68
**p20V10**	27.2	43.6	16.4	0.003**	**0.76**
**p20V13**	21.3	35.0	13.6	0.005**	**0.74**
**p20V20**	14.7	22.1	7.4	0.033	0.68
**p20V30**	10.5	13.8	3.3	0.213	0.60
**p20V40**	7.5	8.9	1.3	0.520	0.56
**p20V50**	4.5	4.8	0.3	0.797	0.55
**p20MLD (cGy)**	987	1310	323	0.052	0.67

AUC, area under the curve; MLD, mean lung dose; ROC, receiver operating characteristic.

Results that reach statistical significance after application of the Bonferroni correction (significance level 0.05/8 = 0.00625) are marked with**.

**Figure 3.**
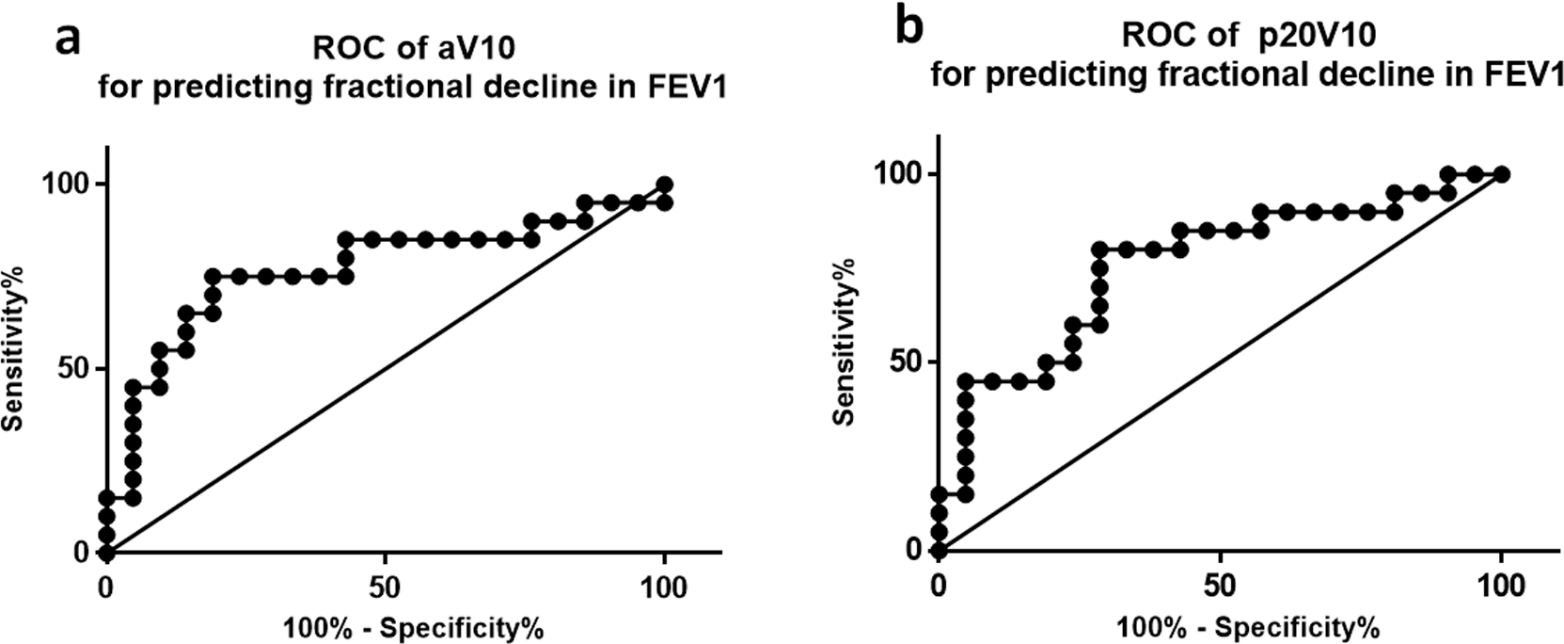
ROC of aV10 (dose distribution within anatomic lung, (a) and pV10 (perfused lung, (b) for predicting decline in FEV1 greater than the median. Area under the ROC (Az) = 0.77 in a and 0.76 in b. ROC, receiver operating characteristic.

ROC analysis for the fractional anatomic and perfused lung volumes receiving >10 Gy (aV10 and pV10 respectively) provided the best predictors of decline in FEV1 following radiotherapy, compared with other dose thresholds (Figure 3). The sensitivity and specificity of aV10 for predicting deterioration in FEV1 at a threshold of 32.7% was 75 and 81% respectively (AUC = 0.77); for pV10, a threshold of 33.5% gave a sensitivity of 80% and specificity of 71.4% (AUC = 0.76).

### Effect of pulmonary dose distribution on CTCAE outcome

CTCAE scores at 3 and/or 6 months (measure of radiation pneumonitis) was available in 40 patients, and at *any* stage during the longer 24 month follow-up period (either early phase radiation pneumonitis or late phase pulmonary fibrosis as a measure of RALI) in 44 patients. The latter broader definition of RALI has been used in previous similar analyses in the literature and increased the number of RT toxicity events in our study cohort from *n* = 4 (radiation pneumonitis) to *n* = 6 (RALI).^7^

Of the 40 patients in whom pre-radiotherapy SPECT scanning, RT dose-distribution and CTCAE follow up at 3–6 months were available, four experienced a worst CTCAE grade of 2–5 (radiation pneumonitis) compared with 36 in whom the worst grade did not exceed 1 ("no radiation pneumonitis"). All 4 were Grade 2 pneumonitis (symptomatic and interfering with activities of daily living, but not requiring oxygen). None experienced early phase CTCAE ≥3 within 6 months. There was no significant difference between the early phase "radiation pneumonitis" and "no radiation pneumonitis" groups in the percentage of anatomic (*p* = 0.24) or perfused (*p* = 0.78) lung volumes that received ≥20 Gy (Table 5).

**Table 5.** Summary of p20 functional lung dose-volume parameters in patients classified as RALI (*n* = 6) on CTCAE and those classified as no RALI on CTCAE (*n* = 38), at different radiotherapy dose thresholds (V5-50) and for the MLD

**% p20** **perfused volume**	**"RALI" (*n* = 6)**	**"No RALI" (*n* = 38)**	**% Diff between medians**	**Mann–Whitney** *p-*value	**ROC AUC (Az)**
**p20V5**	55.7	50.9	4.8	0.472	0.60
**p20V10**	40.0	36.1	3.9	0.514	0.59
**p20V13**	30.7	26.0	4.8	0.514	0.59
**p20V20**	22.1	15.9	6.2	0.582	0.57
**p20V30**	18.0	9.8	8.3	0.472	0.60
**p20V40**	12.2	6.5	5.7	0.431	0.61
**p20V50**	6.8	3.5	3.2	0.514	0.59
**p20MLD** **(cGy)**	1356	1028	328	0.431	0.61

AUC, area under the curve; CTCAE, Common Toxic Criteria for Adverse Events; MLD, mean lung dose; RALI, radiation-associated lung injury; ROC, receiver operating characteristic.

**Figure 4.**
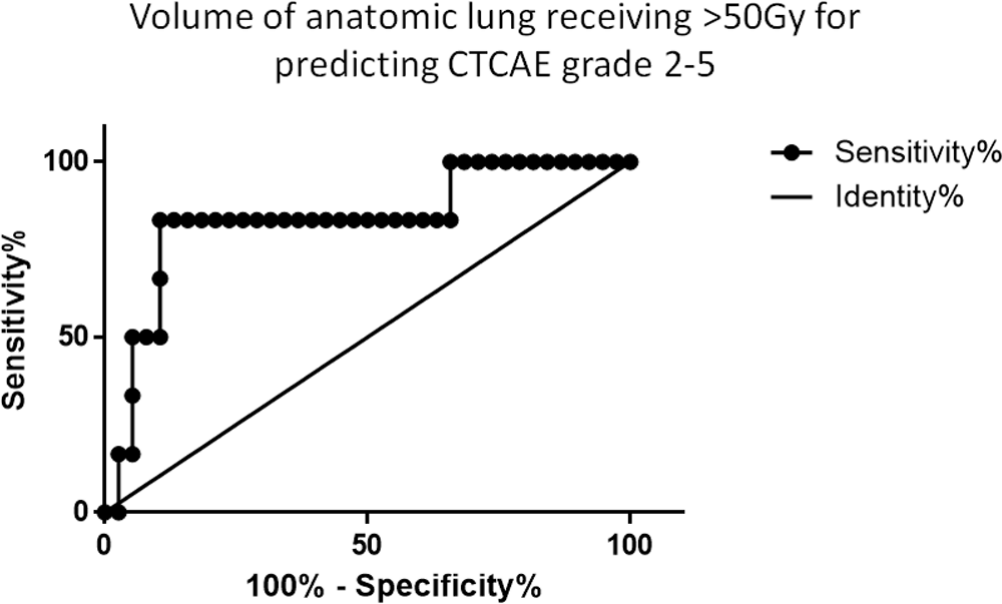
ROC curve of the anatomic lung volume receiving 50 Gy for predicting RALI as assessed by worst CTCAE score at a 3 or 6 month time-point after radiotherapy. CTCAE,Common Toxic Criteria for Adverse Events; RALI, radiation-associated lunginjury; ROC, receiver operating characteristic.

Of the 44 patients in whom SPECT scanning, RT dose-distribution and CTCAE follow up at 0–24 months were available, 6 experienced a worst CTCAE grade of 2–5 ("RALI"), compared with 38 in whom the worst grade did not exceed 1 ("no RALI"). In the six patients with RALI, four had experienced early phase Grade 2 pneumonitis, one experienced late phase CTCAE Grade 3 (50–75% of lung volume fibrotic on imaging), and one late phase CTCAE Grade 5 (death). The perfused lung volumes were not significantly different between the "RALI" and "no RALI" groups at any dose threshold. The fractional volume of anatomic lung receiving ≥50 Gy was significantly different between the "RALI" and "no RALI’" groups. ROC analysis showed that when 4.7% of the anatomic lung volume received >50 Gy, the sensitivity and specificity for predicting subsequent RALI was 83 and 89% respectively (Figure 4).

### Comparison of spirometry with CTCAE

No significant difference in ΔFEV1 following RT was demonstrated between the CTCAE defined "RALI" and "no RALI" groups (Mann–Whitney *p*-value = 0.99). No significant difference was seen between the CTCAE defined "RALI" and "no RALI" groups in terms of either their ∆FVC (Mann–Whitney *p*-value = 0.97) or ∆(FEV1/FVC) ratios (Mann–Whitney *p*-value = 0.52).

Vital capacity and diffusion capacity of lung for carbon monoxide (DLCO) were not evaluated as outcome measures in this study.

## Discussion

The data from our study confirms a significant difference in dose–volume histograms between anatomic (defined on CT) and perfused lung (defined on perfusion SPECT) volumes. This is partly explained by technical differences in the acquisition of lung volume between the techniques (CT in moderate deep inspiration and SPECT in free-breathing), but shows the potential for using perfusion volumes as an independent “organ-at-risk” when determining radiotherapy dose scheduling.

The differences in RT dose to anatomic and perfused lung are in keeping with prior reports, where the feasibility of radiation dose reduction to perfused lung has been demonstrated.^9,26–29^ By retrospectively optimizing RT plans with reference to SPECT perfusion data, one study demonstrated that the greatest dose reduction to perfused lung could be achieved for patients in whom perfusion defects adjacent to tumour provided a potential window for RT delivery, whereas for patients with irregular perfusion scattered throughout both lungs, dose reduction to perfused lung was less achievable.^15^ The volume of perfused lung has also been shown to decrease 1, 2 and 3 weeks after receipt of 15 Gy, indicating that SPECT biomarkers can alert the physician to the likelihood of symptomatic radiation pneumonitis.^30^

Our data also demonstrates that the low integral dose (10–20 Gy) to background lung, defined on either anatomic or by perfusion scans, are the most important dose-scheduling determinants of subsequent decline in FEV1. Therefore, constraining this low integral dose would have the single greatest preserving effect on post-therapy lung function. Prior reports have shown a weak correlation between SPECT-based mean perfusion-weighted lung dose and impairment of both FEV1 and Transfer factor diffusing capacity for carbon monoxide.^18^ Although no randomized controlled trials have yet investigated ability of SPECT perfusion-based RT planning to improve clinical outcome, several observational reports have confirmed its predictive capacity in this regard.^9,14–18,31,32^ Furthermore, the MLD to anatomic but not perfused lung in our study was also significantly higher in the deteriorating FEV1 group and is in keeping with data from other groups.^33^ It may well be that irradiating areas of lung that were under the p20 perfusion threshold (but nonetheless included in the anatomic volume) also affected adverse respiratory outcomes.

The lack of significant difference in our cohort between those classified with or without early phase "radiation pneumonitis" on CTCAE was surprising, in view of data from other groups.^7,14,31^ This is possibly attributable to the fact that only 4 of 40 patients in this study experienced significant "radiation pneumonitis" in the early phase. Farr et al and Hoover et al who showed that including perfused rather than anatomic lung as an "organ-at-risk" improved the ability to predict adverse CTCAE outcome, the proportion of patients with "RALI" was higher in their cohorts (25/58 or 43% and 7/19 = 37% of patients respectively).^7,31^ This could in part be due to the longer follow-up period in their studies (1, 3, 6, 9 and 12 months). However, even with all follow-up time-points for our cohort included (1, 3, 6, 9, 12, 15, 18, 24 months), only 6/44 patients (14%) experienced significant "RALI." As the treatment regimens were equivalent between their datasets and ours (60–66 Gy in 2 Gy fractions), this discrepancy highlights possible differences between institutions in either dose optimization technique or clinical thresholds for grading CTCAE v. 3.0.

From our study, it is noteworthy that the percentage of anatomic lung (but not perfused lung) receiving higher radiation doses (40 and 50 Gy) provided the best predictor of RALI as defined by CTCAE. This suggests that the radiological changes which influence CTCAE score are likely to be the result of the focal high doses delivered to lung surrounding tumour, whereas small airways inflammation that impacts FEV1 is radiologically “invisible.” In line with these findings, ΔFEV1 and CTCAE adverse grade had no relationship in our analysis. Although prior studies have used CTCAE, spirometry or sequential post RT SPECT perfusion scans separately as outcome measures following RT,^7,31,33^ our data records multiple outcome measures in the same study population, which gives an assessment of overall morbidity.

The main limitation of this work arises due to intrinsic anatomic mis-registration between the free-breathing SPECT acquisition and the inspiratory RT planning scans. Free-breathing, perfusion volumes often failed to contact the diaphragms when overlaid on the RT planning volumes, leading to a systematic shift of the perfused compared with the anatomic dose–volume histogram data. The image analysis methods used did not adjust for this. In future, performing a deformable image registration between two sets of CT images (one acquired contemporaneously with the SPECT imaging and the other acquired at RT-planning) should go some way towards mitigating the different levels of inspiration between the techniques.

These results confirm that the fractional volume (anatomic or perfused) of lung that receives >20 Gy differs between patients with subsequent decline in FEV1 but that only the fractional volume of anatomic lung receiving >50 Gy influences the development of RALI as assessed by CTCAE. In patients in whom lung function is limited at the outset, consideration must therefore be given to the percentage of anatomic and perfused lung receiving low doses of radiation. Adding SPECT perfusion imaging as an adjunct to standard CT-based anatomical volumes has the potential to refine RT dose–volume schedules, and to further mitigate RT-induced morbidity.
